# P-424. Challenges in Developing a Non Ventilator Hospital-Acquired Pneumonia Surveillance System

**DOI:** 10.1093/ofid/ofae631.625

**Published:** 2025-01-29

**Authors:** stephen J Cooper, Katherine Fagan, Kelly Cawcutt

**Affiliations:** University of Nebraska Medical Center, Omaha, Nebraska; Nebraska Medicine, Omaha, Nebraska; University of Nebraska Medical Center, Omaha, Nebraska

## Abstract

**Background:**

Pneumonia has been identified as the leading cause of hospital-acquired infections (HAI) with Non Ventilator Hospital-Acquired Pneumonia (NVHAP) making up the majority of cases. The ambiguity of definitions and subjective diagnostic criteria make defining and tracking NVHAP cases a challenge. We attempted to duplicate a previously validated electronic medical record (EMR) based NVHAP surveillance systems at a Midwest Academic Medical Institution.

Defining NV HAP

**Figure:**
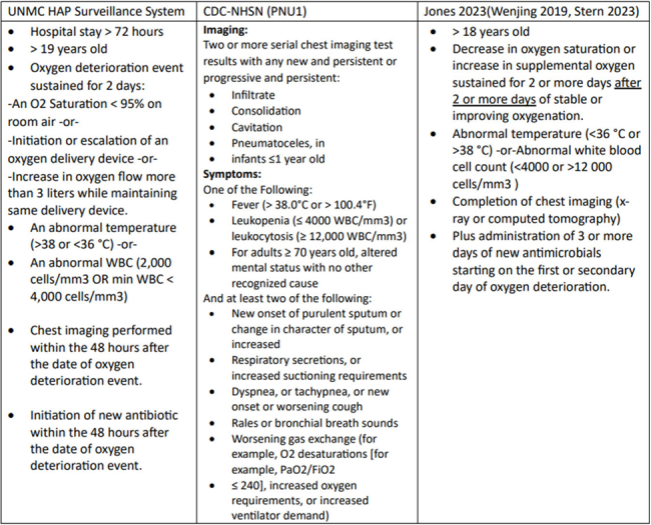

Inclusion criteria for NVHAP.

**Methods:**

Using previously established criteria a surveillance system was designed to determine possible cases of NVHAP using data from our institution’s EMR (Table 1). Institutional review board approval was obtained, and the surveillance system was retrospectively applied to adult patients hospitalized throughout 2023 at two Midwest academic hospitals. A validation study was conducted to compare ten percent of cases reported by the NVHAP surveillance system to that of a single physician reviewer. Interim analysis was conducted in instances of inclusion criteria failure to allow for refinement of the NVHAP surveillance system.

**Results:**

The NVHAP surveillance system generated a list of 425 possible NVHAP events in 2023. Single reviewer analysis of 20 patients revealed 12 patients who did not meet CDC pneumonia criteria and three that did not meet all inclusion criteria: two for lack of chest imaging within 48 hours of recorded oxygen deterioration event and two with no documentation of new antibiotic administration. Additional lists of 338, 322, and 76 NVHAP episodes were generated after coding changes were implemented with repeat interim analysis demonstrating further issues with adherence to inclusion criteria and errors with reproducibility of data extraction.

**Conclusion:**

The development of a NVHAP surveillance system would allow an objective way to track this significant HAI. Potential models exist for creating such a system. However, multiple challenges are still present from determining EMR specific coding for complex medical issues, such as oxygen deterioration events, to reproducibility of electronic extracted data. Our efforts show that even when attempting to replicate validated methodologies site specific challenges will pose significant barriers to the development of EMR based NVHAP surveillance systems.

**Disclosures:**

**Kelly Cawcutt, MD, MS**, BD: Advisor/Consultant|BMJ: Honoraria

